# A Highly Sensitive Detection System based on Proximity-dependent Hybridization with Computer-aided Affinity Maturation of a scFv Antibody

**DOI:** 10.1038/s41598-018-22111-4

**Published:** 2018-03-01

**Authors:** Zhiheng Wang, Yan Li, Wenbin Liang, Junsong Zheng, Shuhui Li, Chuanmin Hu, An Chen

**Affiliations:** 1 Department of Clinical Biochemistry, College of Medical Laboratory, Southwest Hospital, Army Medical University (Third Military Medical University), 30 Gaotanyan Street, Shapingba District, Chongqing, 400038 PR China; 2 Department of Clinical Laboratory Science, College of Medical Laboratory, Southwest Hospital, Army Medical University (Third Military Medical University), 30 Gaotanyan Street, Shapingba District, Chongqing, 400038 PR China

## Abstract

The hepatitis B virus (HBV) infection is a critical health problem worldwide, and HBV preS1 is an important biomarker for monitoring HBV infection. Previously, we found that a murine monoclonal antibody, mAb-D8, targets the preS1 (aa91-107) fragment of HBV. To improve its performance, we prepared the single-chain variable region of mAb-D8 (scFvD8) and constructed the three-dimensional structure of the scFvD8-preS1 (aa91-107) complex by computer modelling. The affinity of scFvD8 was markedly increased by the introduction of mutations L96Tyr to Ser and H98Asp to Ser. Furthermore, a highly sensitive immunosensor was designed based on a proximity-dependent hybridization strategy in which the preS1 antigen competitively reacts with an antibody labelled with DNA, resulting in decreased proximity-dependent hybridization and increased electrochemical signal from the Fc fragment, which can be used for the quantisation of preS1. The results showed a wide detection range from 1 pM to 50 pM with a detection limit of 0.1 pM. The sensitivity and specificity of this immunosensor in clinical serum samples were 100% and 96%, respectively. This study provides a novel system based on proximity-dependent hybridization and the scFv antibody fragment for the rapid quantisation of antigens of interest with a high sensitivity.

## Introduction

Hepatitis B virus (HBV) infection is a prevalent health problem as more than 350 million humans are chronically infected, and nearly one million people die of HBV infection related liver disease every year^1^. To reduce HBV infection complications and mortality, the early and accurate diagnosis and treatment of HBV infection is urgently needed. The traditional serology of HBV infection is diagnostic, and the screening markers are usually referred to as “two pairs of semi-hepatitis B test”. Their main limitation is that they may not accurately reflect HBV replication and viral load. Some of the chronic hepatitis B patients with the HBV gene C region mutations have negative HBeAg test results, but HBV DNA continues to replicate *in vivo*^2^. Based on additional research on HBV preS1 protein, it was found that HBV preS1 test can palliate for the misdiagnosis due to HBeAg deficiency and accurately reflect the virus replication *in vivo*^3,4^. Recent studies have shown that preS1/hepatitis B virus large surface protein (LHBs) detection can be more sensitive than HBeAg and can more accurately reflect HBV replication^5^. The combined application can better reflect the serum HBV virus replication and viral load and minimize the misdiagnosis of HBV gene mutations. At the same time, the HBV preS1 detection can be used to assess the patients’ infection, antiviral therapy efficacy and prognosis^6^. In our previous work, a novel monoclonal antibody specific against HBV preS1, named mAb D8, was obtained using hybridoma technology by immunizing mice with preS1(aa91-aa107) peptide and using HBV preS1 recombinant protein expressed in *E*. *coli* strain BL21 as the screening tool. The sensitivity and specificity of mAb D8 for recognizing recombinant preS1 protein in clinical serum by various methods such as ELISA, Western blot assays and immunocytochemistry has been verified^7^. However, the production of antibody by hybridoma technology is time-consuming, labourious, expensive and of unreliable quality.

With the development of recombinant antibody technology, different derivatives of antibodies and various expression platforms have been reported^8,9^. The single chain variable fragment (scFv), which consists of variable regions of heavy (VH) and variable regions of light (VL) chains of immunoglobulin connected with a flexible linker is the most interesting antibody derivative. ScFv has a small molecular weight, strong penetrating power and weak antigenicity. Additionally, the expression of scFv in mammalian cells or *E*. *coli* BL21 is also stable and efficacious^8,10,11^. However, scFv usually suffers from a low binding affinity. Recently, several methods of mutagenesis by phage display have been shown to be useful for enhancing the affinity of ScFv, in which mutations into the whole gene are introduced by DNA recombination^12^. However, precise control of the degree of point mutation is not possible. Although the hot mutation can limit the mutation to a certain point, the diversity of the mutant library is relatively small. Using these methods, researchers have constructed and screened the mutation library, but the workload is relatively large^13^. The ideal method to study protein-ligand binding mechanism is crystallography, which is a straightforward method for determining the contact residues and accurately guiding the maturation process. Unfortunately, crystallization is not easily completed sometimes^14^. Even if it can be done, it is also an expensive, time-consuming and difficult undertaking. With the increasing number of antibody structures analysed, computer-aided design for antibody *in vitro* affinity maturation is becoming more reliable and convenient. This technology offers a significant advantage over other methods with greatly improved efficacy and success, because it can control the mutation sites within a certain range and target a few or even single amino acid sites^15–17^. Rodrigo Barderas *et al*. constructed the antibody-antigen complex structure model according to the antibody and antigen binding epitope information and obtained an antibody with 450-fold increase in affinity^18^.

At present, the method commonly used for the detection of antigens by single antibody is immune turbidimetric and competitive ELISA. The immunoturbidimetric method requires the use of intact antibodies. The method has a low sensitivity that sometimes cannot meet the clinical detection requirements. The competitive ELISA is a conventional protein detection method based on a single antibody or antigen, but some aspects, such as long detection time, high background, and complexity, still need to be improved. Recently, electrochemical immunosensor with a high sensitivity, fast response, simple operation and low cost has been widely used in life sciences, analytical chemistry and other research areas. Yuan and his co-workers constructed an electrochemical sensor for thrombin detection, which achieved a detection limit of 1 pM^19^. Wang *et al*. used a nano-gold signal amplification to build a sandwich type electrochemical aptamer for platelet-derived growth factor (PDGF-BB) detection, and they achieved an ultrasensitive detection limit up to 0.01 pM^20^. For the sensitive determination of HBV, an electrochemical immunosensor has been reported recently by Rosa F. Dutra *et al*. It was developed for the detection of antibodies to hepatitis B core protein (anti-HBc) with a linear concentration range up to 6 ng/mL and a detection limit of 0.03 ng/mL^21^. Youming Shen and co-workers developed a label-free electrochemical immunosensor based on aldehyde-terminated ionic solution for the determination of hepatitis B surface antigen with a good linear range from 0.05 to 15 ng·mL^−1^ and a detection limit of 20 pg·mL^−1^ ^22^. However, the detection sensitivity and accuracy still need to be further improved. HBV preS1, as a “gold marker” for detection of HBV, has showed great potential for the early diagnosis of HBV. However, until now, no immunosensor for HBV preS1 with an acceptable performance has been reported. In recent years, the immunosensor based on proximity-dependent hybridization strategy (PDHS) is a newly developed DNA-assisted immunoassay with exciting application prospects in the field of trace protein detection^23–25^. Its mechanism relies on the simultaneous recognition of target protein by a pair of antibody or antigen-labelled DNA probes and the hybridization of the proximity-dependent affinity probes. Due to the higher sensitivity and excellent specificity of DNA proximity-dependent hybridization, immunosensors offer a promising alternative for the detection of trace protein or DNA.

Herein, a novel competitive strategy is proposed based on the principle of the proximity-dependent hybridization assay for HBV preS1 detection. First, the Capture Linker hybridized with the ferrocene (Fc) labelled blocking DNA was immobilized on gold nanoparticle (AuNP) modified glassy carbon electrode surface based on the S-Au covalent bond between -SH on the Capture Linker and AuNPs on the electrode surface. The Fc labelling on the blocking DNA could generate obvious strong electrochemical signal in the assay. Based on the proximity-dependent hybridization, the HBV preS1 labelled DNA (Ag-DNA), Associated DNA (As DNA) and HBV preS1 antibody labelled DNA (Ab-DNA) could hybridize with the help of an immune reaction between HBV preS1 and HBV preS1 antibodies. Thus, the Ab-DNA could hybridize with the Capture Linker, releasing the Fc labelled blocking DNA and generating a reduced signal. In the presence of HBV preS1 in the detection system, HBV preS1 would competitively react with Ab-DNA, decreasing the formation of immune complex between the Ab-DNA and Ag-DNA, and more Fc labelled blocking DNA would hybridize with the Capture Linker. Thus, a higher electrochemical signal from Fc would be obtained, which was associated the presence of HBV preS1 in the detection system for the quantitative detection of HBV preS1. Because of the specific hybridization among the capture probe, Fc-probe, Ag-DNA and Ab-DNA at a predesigned melting temperature, the surface proximity assay was achieved. This biosensor was shown to be sensitive, specific, and selective with a low detection limit and a wide linear dynamic range.

In this study, we cloned the variable region genes of mAb-D8 by PCR and phage display, prepared the single chain variable fragment of mAb-D8, and constructed the three-dimensional structure of anti-preS1 scFvD8. The preS1 (aa91-107) peptide was docked to the scFv model. Then, the binding site of scFvD8 was analysed, and the mutant was designed and constructed. Finally, we constructed an immunosensor based on proximity-dependent surface hybridization strategy to achieve rapid and efficient HBV preS1 detection. The scFvD8 mutant (scFvD8-M) we propose here provides an inexpensive, time-saving structure-based rational method to accelerate the development of affinity-matured antibody with enhanced potential for antibody engineering. Additionally, the strategy we provided here to construct a fast, highly sensitive and quantitative detection system based on one scFv and proximity-dependent hybridization strategy is valuable for the detection of other interested proteins.

## Results and Discussion

### Cloning and assembly of VH and VL gene

**Figure 1:**
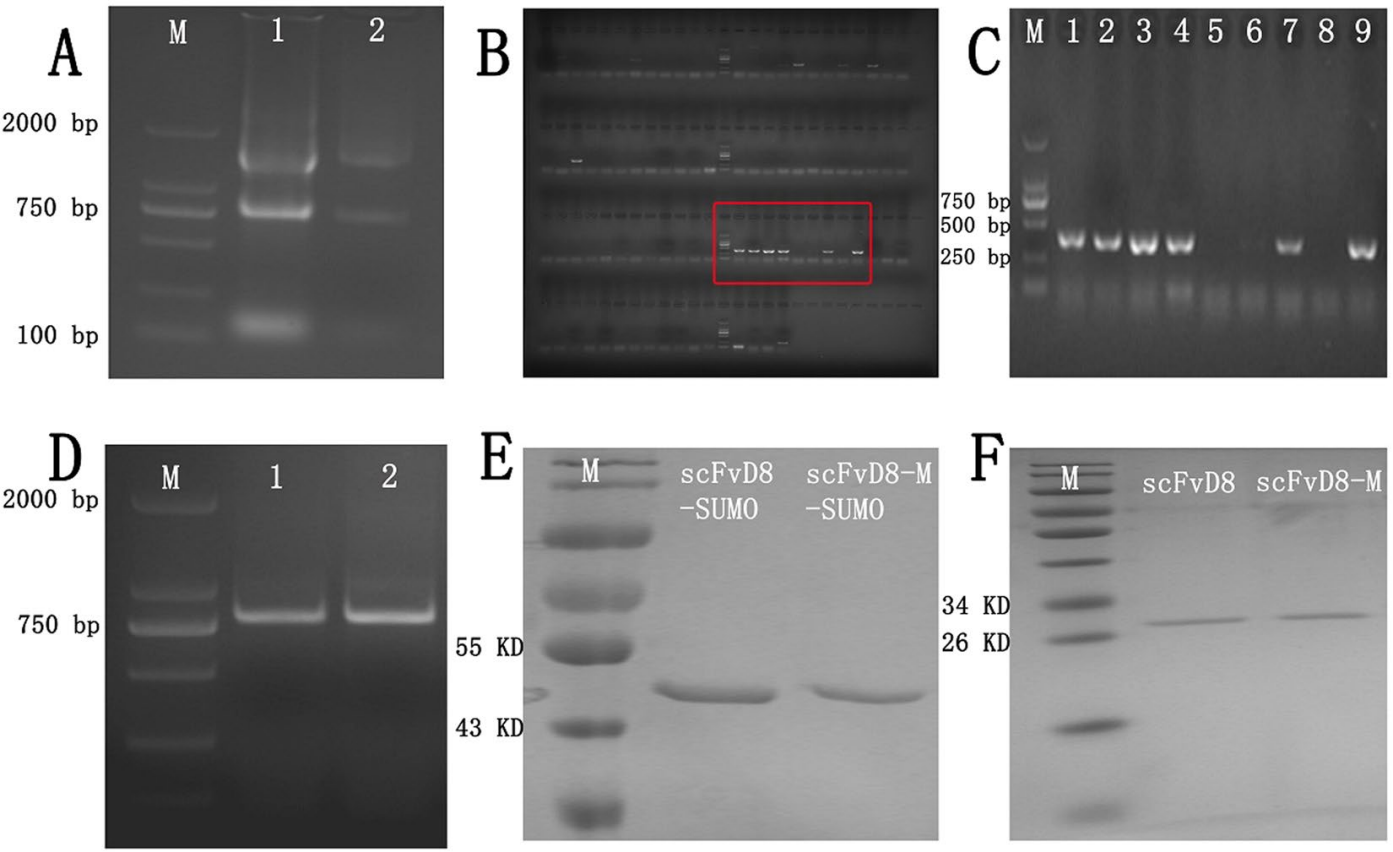
Construction and expression of anti-preS1 scFvD8 and scFvD8-M. (**A**) Total RNA from hybridoma. Lanes: M: marker 2000; 1 and 2: total RNA from mAb D8 hybridoma. (**B**) PCR amplification of VH and VL with 87 pairs of primers. (**C**) Enlarged inset from picture B showing the amplification of VL. (**D**) PCR amplification of scFv gene. Lanes: M: marker 2000; 1 and 2: the scFv fragment of anti-preS1 D8 assembled with a (G_4_S_1_)_4_ linker. (**E**) SDS-PAGE identification of the purified protein, which was expressed with the pET28a-SUMO vector in the BL21 host. (**F**) SDS-PAGE identification of the purified protein, which was expressed with the pcDNA3.4 vector in CHO-S cells.

The monoclonal antibody, mAb-D8, directed against preS1 (aa91-107) of the large hepatitis B surface antigen was purified from mouse hybridoma cell culture. In our previous work, it was confirmed that this mouse monoclonal antibody could be employed for the detection of clinical samples. To obtain the gene encoding the antibody variable region, total RNA was isolated from the hybridoma cell line secreting mAb-D8 (Figs 1A and S1) and reversed transcribed into cDNA. A total of 87 pairs of mouse antibody gene primers to amplify the cDNA, in which 5 pairs of heavy chain primers successfully amplified the expected size DNA fragment between 250 bp and 500 bp, and 6 pairs of light chain primers amplified the corresponding size DNA fragment (Figs 1B,C and S2). Light-chain and heavy-chain genes were re-amplified by overlapping PCR, adding two restriction sites and one (G_4_S_1_)_4_ linker to generate the final scFv gene (Figs 1D and S3).

### Anti-preS1 scFvD8 panning and DNA sequencing

**Figure 2:**
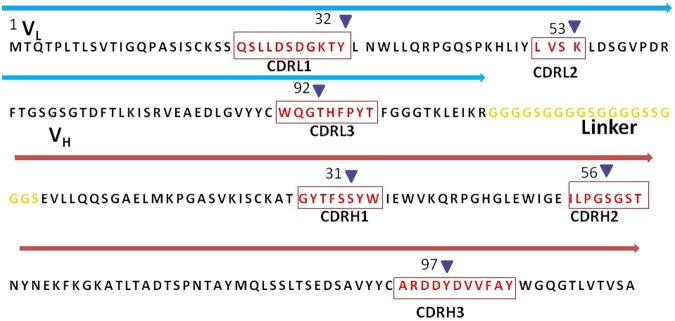
Amino acid sequence of scFvD8 from VL (below the light blue arrow) and VH (below the brown arrow). The linker region is indicated in yellow. CDR regions are highlighted in red. The binding site of scFvD8 was boxed under the “” symbol with Kabat numbering.

After three rounds of panning, 88 clones were selected for phage ELISA and 32 positive clones were obtained (OD_positive_/OD_negative control_ > 5). Ten clones with high OD were selected for sequencing. The sequencing results showed that 8 clones were identical and could be translated correctly. The greatest advantage of the phage display methods is the ability to obtain functional antibodies directly and avoid invalid, nonfunctional or aberrant gene interference. Therefore, the phage display method was chosen over the direct cloning of the gene into an expression vector^26^. The DNA sequencing results revealed that the clones possessed the basic characteristics of immunoglobin variable region genes. We selected a clone with many copies and the highest affinity as our ultimate gene of interest. The CDR regions were determined according to IgBLAST analysis (Fig. 2).

### Expression of anti-preS1 scFvD8 in BL21 (DE3) and CHO-S cells

A number of prokaryotic expression vectors, such as pET22b, pET28a, and pET32a, were employed to express scFvD8 and its mutant in BL21 host. However, the scFv recombinant protein was rarely expressed or expressed at a low level of inclusion bodies. The Small Ubiquitin-like modifier (SUMO) protein as a fusion tag and molecular chaperone was inserted into the vector pET28a for recombinant protein expression; this insertion could not only further improve the expression of the fusion protein but also contribute to protease hydrolysis, proper folding and improvement of the solubility of the recombinant protein^27,28^. Although we still failed to achieve the soluble expression, the protein expression level increased, rising to 100 mg/L under optimized conditions (20 °C, 0.1 mM IPTG and overnight expression). The product was subjected to slow gradient pH dialysis in the TGE buffer (Figs 1E and S4). Transfection of the eukaryotic expression vector pcDNA3.4-scFvD8(D8-M) into the mammalian cells CHO-S using liposomes successfully achieved an expression level of 40 mg/L of the biologically active protein without renaturation (Figs 1F and S5). The expressed protein was purified by His affinity chromatography and identified by SDS-PAGE gel electrophoresis.

### Homology model building and docking

**Figure 3:**
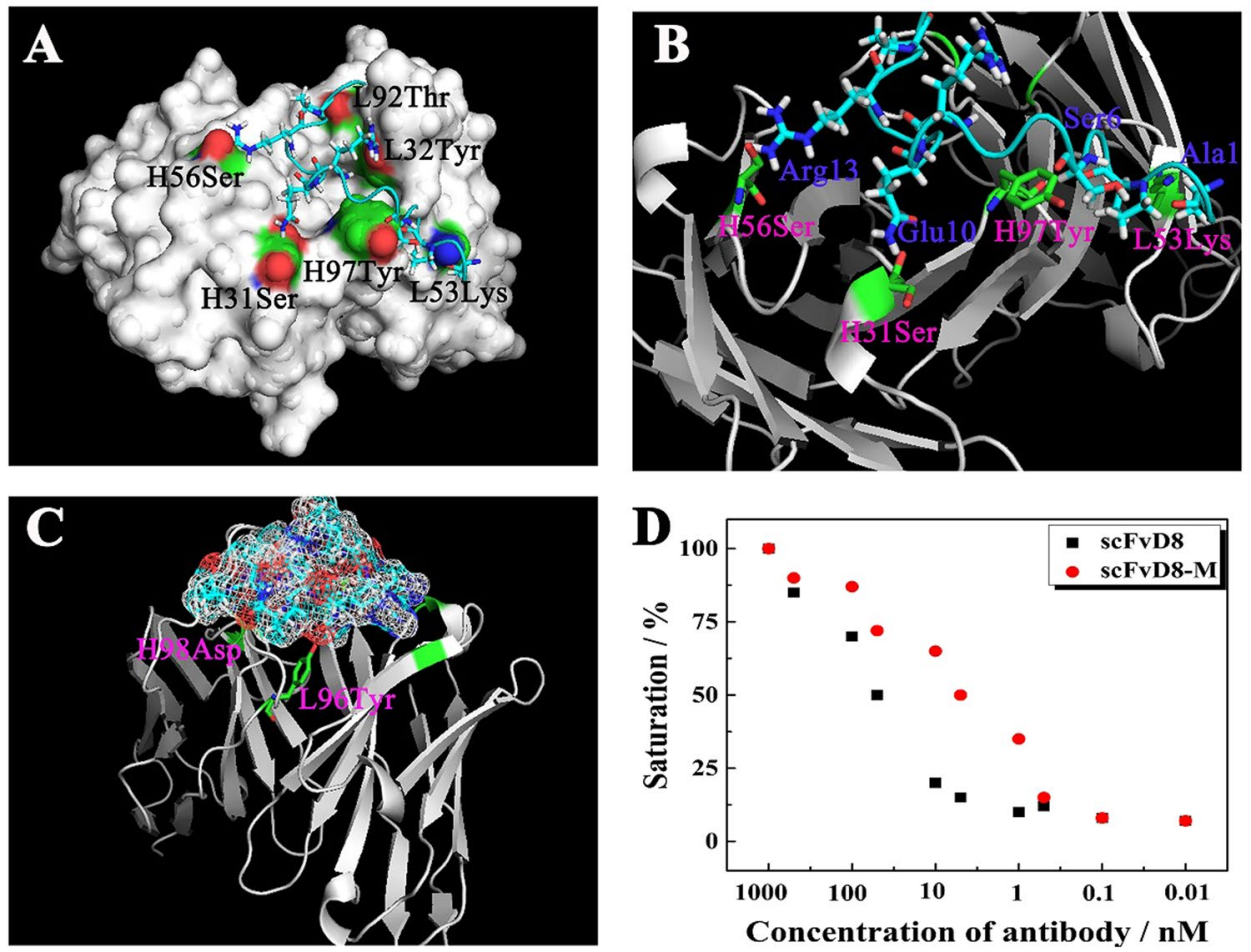
Binding complex of scFvD8 and preS1 (aa91-107). (**A**) Pocket of scFvD8 and preS1 (aa91-107) interaction. (**B**) Docking model of the amino acid residues of preS1 (aa91-107) inside the binding pocket of scFvD8. The distance between interacting residues is not greater than 0.3 nm with the opposite charge. (**C**) Point mutation site interaction interface. The model of preS1 (aa91-107) shows the surface electrostatic potential. The positive charge region is shown in red, and the negative charge region is in green. The positively charged amino acid residue L96Tyr of scFvD8 falls into the positive charge region of preS1 (aa91-107). (**D**) The relative affinities of scFvD8 and scFvD8-M were measured by competitive ELISA. The concentrations of preS1 recombinant protein were 1000, 500, 100, 50, 10, 5, 1, 0.1 and 0.01 nM. At 50% protein binding saturation, the concentration of scFvD8 was 50 nM, and the concentration of scFvD8-M was 5 nM.

The structural model data obtained by MODELER are considered credible when the homology to the reference protein is equal to or greater than 40%. As more and more antibody structures have been resolved, the likelihood of obtaining such homologous structures in the PDB database has increased. The results showed that the template (PDB: 1d5i) homology for the heavy chain is 88.9% and the template (PDB: 1nld) for the light chain is 96.5%. The high homology ensures the accuracy of the later modelling. Additionally, it is comparatively easier to analyse the antigen-antibody binding site because the peptide preS1 (aa91-107) peptide has only 17 amino acids. According to the c-core data, the most reliable peptide model was selected to dock. After docking, a complex structure model was obtained, and the distance of the amino acid residues between scFvD8 and preS1 (aa91-107) peptide was calculated (Fig. 3A). The amino acid sites of preS1 (aa91-107) interacting with the antibody-binding region were determined based on the distance, which is not greater than 0.3 nm. The structure and properties of these amino acids were analysed and are summarized in Table 1.

**Table 1:** The interacting amino acid residues between preS1 (aa91-107) and scFv-D8.

PreS1 (aa91-107) peptide residue	ScFv residue	Location	Distance (nm)
Arg13	H56Ser	CDRH2	1.63/0.52
Ser6	H97Tyr	CDRH3	2.45
Gln10	H31Ser	CDRH1	1.76
Ala1	L53Lys	CDRL2	1.45
Pro4	L32Tyr	CDRL1	1.87
Pro5	L92Thr	CDRL3	2.13
Arg9	L32Tyr	CDRL1	2.84

Antibody scFvD8 Light chain-Lys53, Tyr32, and Thr92 are adjacent to the residues of preS1 (aa91-107)-Ala1, Pro4 and Arg9, and Pro15, respectively. These amino acid residues carry the opposite charge, and the distance between them is less than 0.3 nm, indicating that they may form hydrogen bonds. At the same time, scFvD8 heavy chain-Ser56, Tyr97, and Ser31 are adjacent to the residues of preS1 (aa91-107)-Arg13, Ser6, and Gln10 (Fig. 3B). One oxygen atom on Ser56 of the heavy chain falls between two hydrogen atoms on the residue Arg13 of the preS1 (aa91-107), and the distances between the oxygen atoms and the two hydrogen atoms are 1.63 nm and 0.52 nm. The site of the combination is the same as the anchor, and thus, the antibody firmly grasps the antigen.

### Point mutations of scFv and effect on affinity

The interaction force between molecules mainly depends on hydrophobic interactions, hydrogen bonds, and ionic bonds. The bond energy of hydrophobic interactions is approximately 1~2 kJ/mol, the hydrogen bond force is 1 to 2 times greater, and the ionic bond is the strongest, approximately one order of magnitude higher^29^. Through in-depth analysis of the interface residues, the local environment of the residues can be understood and improved by changing some of the amino acid sites. According to the interaction between preS1 (aa91-107) and scFvD8, the following conclusions can be drawn. The residue L96Tyr is close to and carries the same charge as the amino acid residue Gly12 of preS1 (aa91-107), and the residues of L96Tyr act like a bridge located between the antibody and peptide binding sites. Mutating this amino acid may increase the antigen-antibody interaction area (Fig. 3C). The Asp98 of the heavy chain and the preS1 (aa91-107) of the amino acid residues Pro4 are close to each other and carry the same charge, and the two mutually interacting sites are located at the antigen-antibody binding interface. It is possible to remove the negative charge of the Asp98 to change the local charge environment or to add a positive charge at that site to form an ionic bond to increase the antibody and antigen interaction force.

A mutant was identified by PyMol observation of the interaction between the structural interface of the complex and the interaction of the amino acid residues. The 3D model of the mutant was constructed by homology modelling, and the changes of free energy before and after mutation were analysed. FoldX is a molecular modelling and protein design software programme that can be run in YASARA for analysing the protein-protein interaction energy and calculating the difference of free energy between the designed mutant and parent antibody, according to ∆∆G (change) = ∆G (MT) − ∆G(WT), where the ∆∆G value is close to the experimental value. If ∆∆G is negative, the mutant is more stable than the parent antibody because of the reduced potential energy. A positive value would indicate that the structure is unstable^30^. The scFvD8 structure and the mutation site were uploaded in Foldx using “Mutate multiple residues” to analyse the change of ∆∆G before and after the scFvD8 mutation and to calculate the difference. When two sites undergo such amino acid mutations, L96Tyr to Ser, and H98Asp to Ser, the difference in free energy is optimal, ∆∆G = −5.41463 kcal/mol. Therefore, the mutant is more stable than the parent antibody.

### The design of electrochemical sensor

**Figure 4:**
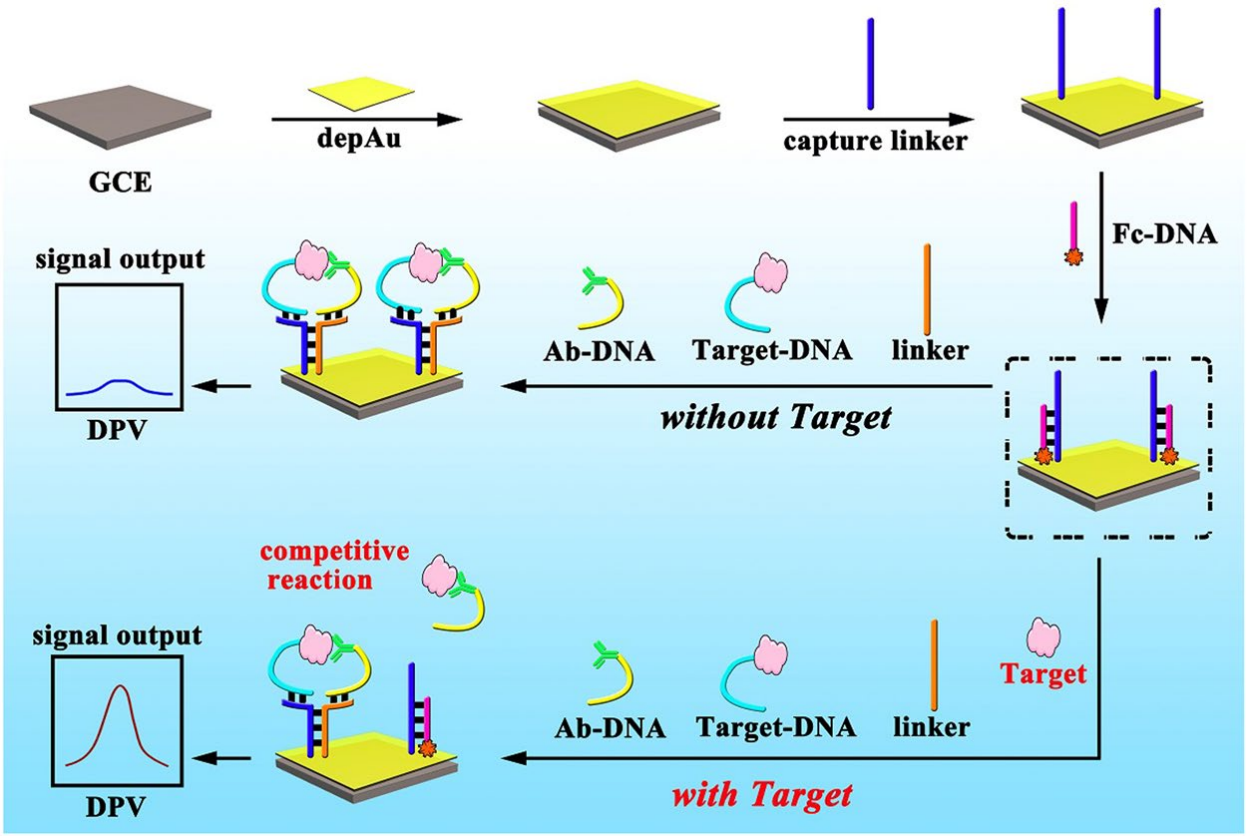
Detection strategy based on the proximity-dependent hybridization.

The schematic representation of the proposed electrochemical sensor based on proximity-dependent hybridization strategy is shown in Figs 4 and S7. The oligonucleotides which were designed to be used in this work are listed in Table 2. First, the cleaned glassy carbon electrode was modified with gold nanoparticles (AuNPs) to immobilize the Capture Linker hybridized with the ferrocene (Fc) labelled blocking DNA. The latter was modified onto the electrode surface by the S-Au covalent bond between –SH on the Capture Linker and AuNPs on the electrode surface. A clear electrochemical signal of Fc will be produced due to its electrochemical oxidation-reduction reactions on the electrode surface. The Capture Linker can hybridize with Ag-DNA and As DNA with the help of the immune reaction between HBV preS1 and HBV preS1 antibodies. Based on the proximity-dependent hybridization, the hybridized products of As DNA and Ab-DNA could hybridize with the Capture Linker, releasing the Fc labelled blocking DNA and generating a reduced signal *via* the electrochemical reaction of Fc. In the presence of dissociating HBV preS1 in the samples, it could react with Ab-DNA and decrease the reaction efficiency of the proximity-dependent hybridization, producing a decreased electrochemical signal by Fc. Thus, a reduced formation of DNA-Ag-Ab-DNA complex with a detectable electrochemical signal enhancement would depend on the concentration of dissociating HBV preS1 antigens in the samples, which could be used for the quantization of HBV preS1.

**Table 2:** Sequences of the oligonucleotides used in this work.

Oligonucleotides	Oligonucleotide sequences
Capture Linker	5′-SH-GCA TGA ATT TTC GTT CGT TAG GGT TCA AAT CCG CG-3′
AgArm	5′-NH2-C6-CCC ACT TAA CCT CAA TCC ACG CGC GGA TTT GAA CCC TAA CG-3′
AbArm	5′-TAG GAA AAG GAG GAG GGT GGC CCA CTT AAA CCT CAA TCC A-C6-NH2-3′
Associated DNA	5′- CCA CCC TCC TCC TTT TCC TAT CTC TCC CTC GTC ACC ATG C- 3′
Competitor DNA	5′-CGT TCA TGC-Fc-3′

### Characteristics of the proximity-dependent hybridization biosensor

**Figure 5:**
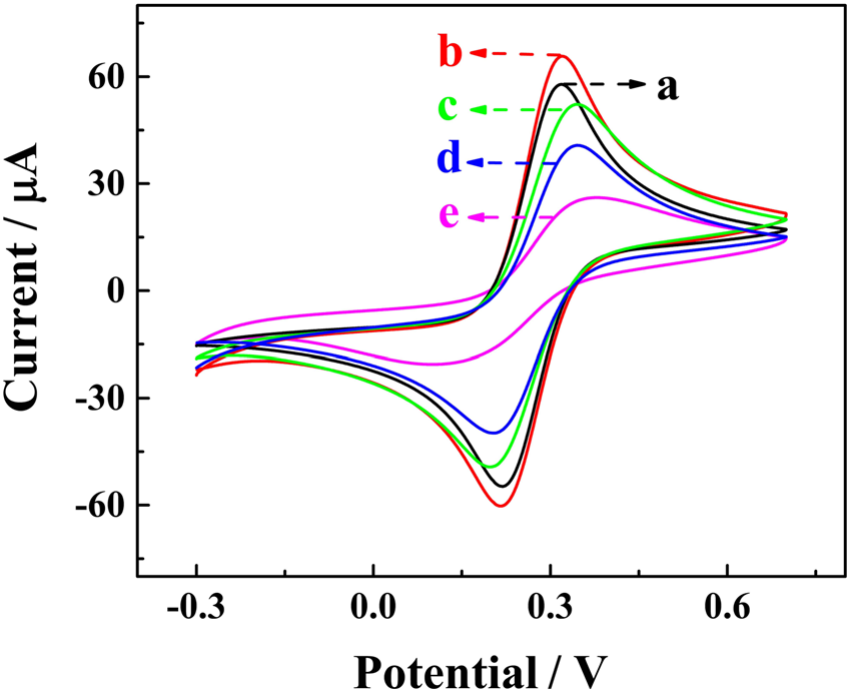
The CV characteristics of the electrode assembly process in the presence of buffer solution (0.1 M KCl and 2.5 mM Fe(CN)_6_^4−/3−^). The labels denote: a, blank glassy carbon electrode; b, after AuNP modified glassy carbon electrode; c, after the modification of the Capture Linker; d, after the hybridization of the Fc labelled blocking DNA; e, after blockade with HT.

Cyclic voltammetry (CV) experiment is a conventional method for studying the electrochemical properties of sensors. As shown in Fig. 5, the CV of the electrode assembly process was characterized in the presence of buffer solution (0.1 M KCl and 2.5 mM Fe(CN)_6_^4−/3−^). The curve a showed the CV of the blank glassy carbon electrode with a pair of Fe(CN)_6_^4−/3−^ redox peaks. When the gold nanoparticles were modified on the electrode surface, the CV redox peak current was significantly enhanced due to the enhanced surface areas and the excellent conductivity of nano-Au. After the modification of Capture Linker on the electrode surface, the CV redox peak current is significantly reduced due to the inherent inertia of the Capture Linker (curve c). After the hybridization of the Fc labelled blocking DNA to the Capture Linker sequences, the CV redox peak current is slightly further reduced (curve d). When the proposed electrode was blocked with 6-mercapto-1-hexanol (HT) to reduce the non-specific adsorption site (curve e), a decreased peak current could be observed due to the closed performance of the HT.

### Optimization of experimental conditions

**Figure 6:**
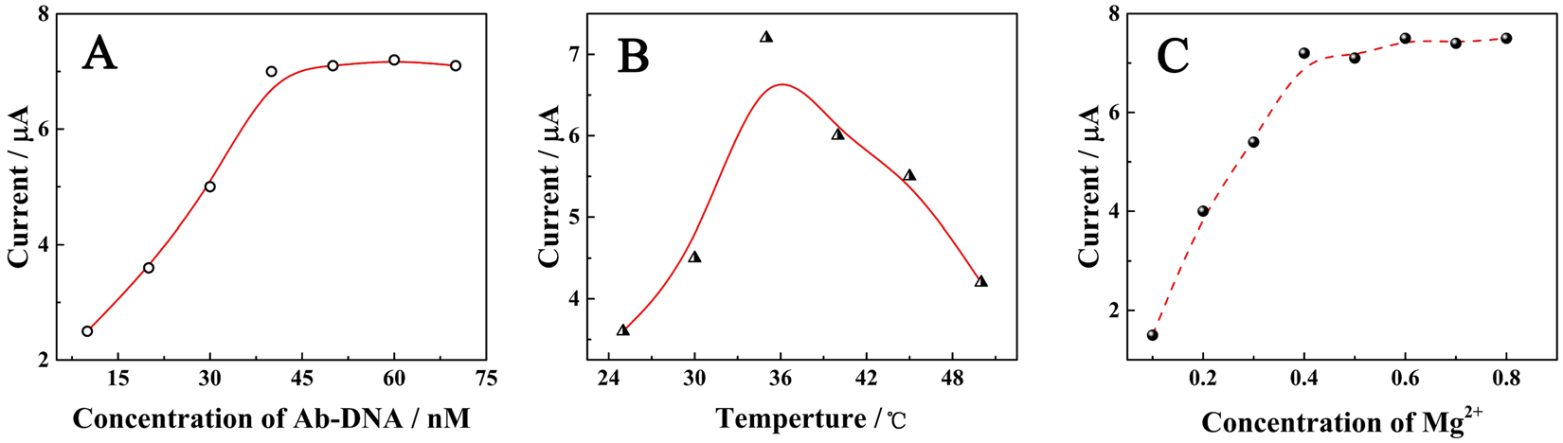
The optimization of DNA hybridization conditions. (**A**) Effects of the Ab-DNA/Ag-DNA concentration (10 to 70 nM) on the detection of 50 pM preS1 recombinant protein. Reaction conditions: TBE buffer (pH 7.4), 0.4 M Mg^2+^, incubation at 37 °C for 1 h. (**B**) Effects of temperature (25 to 50 °C) on the detection of 50 pM preS1 recombinant protein. Reaction conditions: 50 nM Ab-DNA/Ag-DNA, TBE buffer (pH 7.4), 0.4 M Mg^2+^, incubation for 1 h. (**C**) Effects of the Mg^2+^ concentration (0 to 0.8 M) on the detection of 50 pM preS1 recombinant protein. Reaction conditions: 50 nM Ab-DNA/Ag-DNA, TBE buffer (pH 7.4), incubation at 37 °C for 1 h.

DNA hybridization was influenced strongly by the reaction conditions, such as the DNA hybridization concentration, the experimental temperature and the concentration of Mg^2+^ in the reaction buffer^31,32^. First, the appropriate concentration of Ab-DNA/Ag-DNA was determined. Figures 6A and S6A shows the current response of the proposed immunosensors at different Ab-DNA/Ag-DNA concentration in the presence of 50 pM preS1 recombinant protein. The current response value continuously increased up to 50 nM and then reached a plateau, indicating that specific recognition had reached saturation. The appropriate concentration of Ab-DNA/Ag-DNA was set at 50 nM.

The effect of temperature was examined between 25 °C and 50 °C, as shown in Figs 6B and S6B. The current response increased gradually with increasing temperature and reached its maximum at 35 °C. Thereafter, the signal gradually fell, possibly because the high temperature denatured the DNA strands. Thus, 35 °C were adopted as the optimum hybridization temperature.

Figures 6C and S6C shows the effect of Mg^2+^ concentration from 0 to 0.8 M on the electrochemical readings of DNA biosensors. With increasing Mg^2+^ concentration to 0.4 M, the current response increased significantly, reflecting the improvement of the hybridization performance, and then reached a plateau. With the increase of Mg^2+^ concentration, DNA hybridization non-specific reaction was also enhanced. Therefore, 0.4 M was chosen as the most appropriate concentration of Mg^2+^.

### The performance of the proposed electrochemical immunosensor

**Figure 7:**
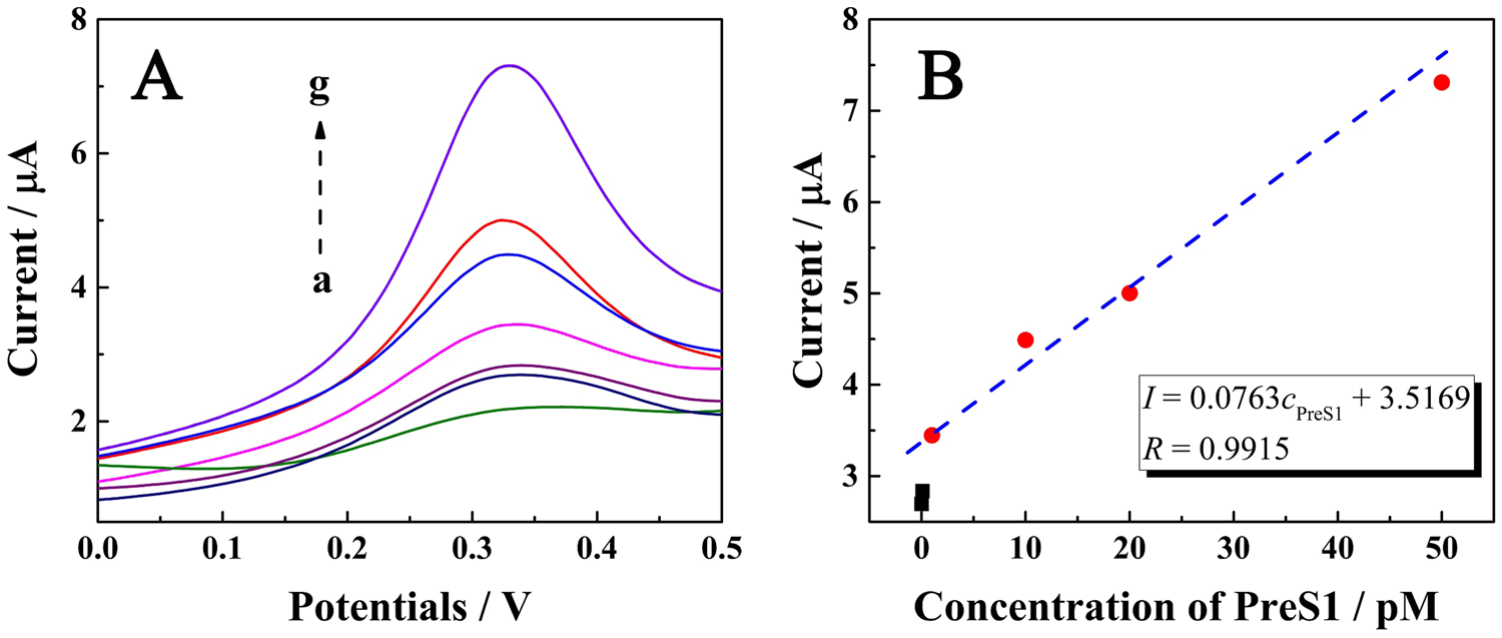
Electrochemical signals as a function of the concentration of preS1 recombinant protein. (**A**) Responses of the electrochemical biosensor at different concentrations of preS1 recombinant protein (curves a-g, at concentrations of 0, 0.01, 0.1, 1, 10, 20, and 50 pM, respectively). (**B**) Calibration curve for the relationship between electrochemical signal and concentration of preS1 recombinant protein.

The analytical characteristics of the proposed immunosensor were evaluated with different concentrations of preS1 recombinant protein. As shown in Fig. 7, the DPV signals increased as the concentration of preS1 increased with a linear dose-response curve in the range from 1 pM to 50 pM with a detection limit of 0.1 pM. Further studies for the detection of preS1 in a clinical serum matrix were performed by the standard addition method. The concentration range of preS1 recombinant protein was consistent with that measured in PBS, and the results of the electrochemical assay were consistent in both matrix solutions, indicating that the proposed immunosensors were capable of detecting preS1 recombinant protein in serum and had the potential to perform well with clinical samples. A comparison of different biosensors for the detection of HBV was listed in Table 3.

**Table 3:** Comparison of different biosensors for the detection of HBV.

Detection Methods	Detection Target	Linear Range	Detection Limit	Ref.
ECA	HBV DNA	0.4–10 nM	0.01 nM	^41^
ECA	HBsAg	1 ng –10 µg mL^−1^	0.14 ng mL^−1^	^42^
ECA	HBsAg	0.05–15 ng mL^−1^	20 pg mL^−1^	^22^
ECA	anti-HBc	1–6 ng mL^−1^	0.03 ng mL^−1^	^21^
ECLA	HBV DNA	0.5 pM–0.5 nM	0.082 pM	^43^
ECA	HBV preS1	1 pM–50 pM	0.1 pM	This study

Abbreviations: ECA, electrochemical assay; ECLA, electrochemiluminescence assay; HBsAg, hepatitis B surface antigen; anti-HBc, antibodies to hepatitis B core protein.

The current responses of 10 pM preS1 recombinant protein without or with potentially interfering proteins IgG, neutrophil gelatinase associated lipocalin (NGAL), procalcitonin (PCT), or rheumatoid factor (RF) at a concentration of 100 pM were further investigated. No significant difference in current with other protein solutions were observed compared to the current in the presence of only preS1 recombinant protein, even though the concentration of interfering proteins was much higher than that of preS1 recombinant protein, indicating the excellent selectivity of the proposed immunosensor.

To verify the practicability of the immunosensor, we collected 50 clinical serum samples from the Southwest Hospital (Chongqing, China) for a double-blind test, including 35 HBV preS1 positive samples and 15 negative samples identified by ELISA qualitative test (Shanghai Fosun Long March Medical Science Co., Ltd.). For the 35 positive samples, 35 were found to be positive (100% sensitivity), and for the 15 negative samples, 13 samples were identified as negative based on our proposed immunosensor, with a specificity of 96%, supporting the potential of our immunosensor for clinical application for the early diagnosis of HBV infection.

### Reproducibility and stability

The reproducibility of the proposed immunosensor was evaluated based on the inter- and intra-assay at different times and with different batches of immunosensors testing the same standard solution. A standard deviation less than 4% was obtained, indicating the excellent reproducibility of the proposed immunosensors. As one of the key to the performance of immunosensor, their stability was estimated based on the long-term storage assay. Within a period of 2 weeks, the proposed immunosensors maintained an initial reaction of 92.21%, indicating the acceptable stability of the proposed immunosensor.

## Conclusions

In conclusion, the variable region gene of the mAb-D8 that recognizes HBV preS1 was obtained by phage display technology, a highly similar homology model was constructed, and the preS1 (aa91-107) peptide was docked to the model. By means of the auxiliary analysis software, the binding site was determined, which was composed of H56Ser, H97Tyr, H31Ser, L53Lys, L32Tyr, and L92Thr. Two amino acids were mutated: L96Tyr to Ser and H98Asp to Ser. The mutant relative affinity assayed by ELISA using thiocyanate elution confirmed a 10-fold increase compared to scFvD8. This work lays the foundation for further improving the affinity of anti-preS1 using the mutant as a template to continue to optimize the antigen-antibody interaction interface. An immunosensor based on PDHS for rapid quantisation was constructed to detect preS1 recombinant protein with a linear dose-response curve in the range of 1 pM to 50 pM. The proposed immunosensors were evaluated with regard to their sensitivity, specificity, reproducibility and stability. All the characteristics support the great potential for HBV preS1 rapid quantitative detection and commercial application. Importantly, the success in establishing a detection system based on one scFv antibody offered a simple and efficient strategy for detecting various targets, which provides a new avenue for infectious disease surveillance, food safety monitoring and clinical diagnostics.

## Methods

### Synthesis of mAb D8 VL and VH regions

Mouse hybridoma cell line D8 that secretes monoclonal antibody against HBV preS1 was generated from splenocytes of mice immunized with peptide preS1 (aa91-107). The DNA sequences encoding theVL and VH chains were obtained according to the method and procedure described by Robert Aitken^33^. Briefly, total RNA was isolated from the hybridoma cell line D8 using Trizol reagent according to the manufacturer’s protocol. The cDNA was synthesized using a reverse transcriptase kit with total RNA as template and oligo (dT) 15 primer. The resulting cDNA was used to amplify VH and VL fragments using Q5 High-Fidelity DNA polymerase (NEB) and recombined by a second round of PCR with primers as described by Robert Aitken. Thereafter, VL and VH fragments were assembled into scFv by overlapping PCR using external primers. Finally, the PCR products were purified by gel electrophoresis, digested with *Sfil* and *BamHI* restriction enzymes, and ligated into the phagemid vector pComb3xss. Escherichia coli OMINImax cells were electroporated (2.5 kV, 25 µF, 200 Ω) with the ligation mixture using Gene Pulser II (Bio-Rad Laboratories, Munich, Germany). Library phages were harvested from the culture supernatant of recombinant *E*. *coli* and precipitated with 20% PEG, 2.5 M NaCl. The phage pellet was reconstituted in PBT buffer and 10% glycerine.

### Anti-preS1 ScFvD8 panning and DNA sequencing

The scFv of D8 antibody (scFvD8) was panned as described by Robert Aitken^26^. First, the immunotubes were coated with recombinant HBV preS1 at 5 µg/L in coating buffer overnight at 4 °C and blocked with blocking buffer for 2 h at room temperature. After rinsing with PBST buffer, the library phage was diluted to one tenth with blocking buffer; 1 mL was added to the antigen-coated immunotube, and incubated for 2 h at room temperature with shaking (100 rpm). Phages were eluted with 100 mM HCl and used for infection of the exponentially growing OMINImax. After 3 rounds of panning, phage ELISA was performed to identify the individual colonies. Plasmids of positive clones were prepared and sequenced.

### Molecular modelling of scFvD8 and preS1 (aa91-107) peptide

The sequences of VL and VH were analysed using online V-Quest software provided by the international ImMunoGeneTics database (IMGT)^34^. A scFv homology model was built using the Prediction of ImmunoGlobulin Structure (PIGS) server according to the structure of PDB: 1d5i (sequence identity 88.9) as the template for heavy chain and PDB: 1nld (sequence identity 96.5) as the template for light chain^35^. The molecular model of preS1 (aa91-107) peptide was predicted using I-TASSER^36^. The server generated five models and the best one was selected based on the C-score. All the predicted models were viewed and analysed in Pymol viewer^37^.

### Molecular docking of the preS1 peptide and scFvs

Web based server ‘PatchDock’ was used for the peptide-scFv docking studies^38^. Molecular models of scFv and preS1 peptide were used as input for the antigen-antibody docking algorithm. Twenty models were generated by the server, and the top ten solutions with a near-native conformation were identified. The server ranked the models on the basis of geometric score, desolvation energy, interface area size and the actual rigid transformation of the solution. The highest ranked model was selected for analysis.

### Computational design of mutants using FoldX

The computational design of scFvD8-M was performed using FoldX version 3.0^39^. According to the molecular docking structure model, the interface residues were identified, and two mutant sites, which might affect the affinity, were identified. The specific residues were mutated based on the scanning of the mutant residues in FoldX with the other 19 naturally occurring amino acids. The change in the binding free energy (G, kcal/mol) of the protein-protein complex was calculated and the best amino acid mutation was selected according to the significance of its reduction in binding free energy.

### Cloning of anti-preS1 scFvD8 mutants

Point mutations were created in scFvD8 sequence to synthesize scFvD8-M using Q5 Site-Directed Mutagenesis Kit (NEB) according to the manufacturer’s instructions. The primers were designed using NEB online software. The gene of scFvD8 serving as the template were cloned in phagemid vector pComb3xss with *EcoRI* restriction site on N-terminus and with His-tag and *XhoI* restriction site on the C-terminus. All the scFvD8 mutants were transformed into *E*. *coli* DH5a cells on LB Agar plates and selected by kanamycin resistance. Plasmids were isolated and tested for mutation by PCR amplification, restriction digestion and DNA sequencing.

### Expression and purification of anti-preS1 scFvD8 and scFvD8-M

First, the prokaryotic expression vector pET28a-SUMO was constructed by inserting SUMO protein to the N-terminus of the reading frame of the vector pET28a. The antibody gene for scFvD8 and scFvD8-M was then digested by restriction enzymes *XhoI* and *EcoRI* and ligated into vector pET28a-SUMO with T4 DNA ligase. The recombinant plasmid pET28a-SUMO-scFvD8 and pET28a-SUMO-scFvD8-M were transformed into *E*. *coli* BL21 (DE3) cells, and the expression was followed using normal procedures. For an efficient production, different conditions of temperature (37 °C, 32 °C, 28 °C, 22 °C and 18 °C), different IPTG concentrations (0.1 mM, 0.2 mM, 0.4 mM, 0.6 mM, 0.8 mM and 1 mM) and induction times (4 h and overnight) were tested. The inclusion body renaturation was achieved by slow gradient pH dialysis in the TGE buffer.

The construction method of the eukaryotic expression vector pcDNA3.4-scFvD8 (M) was as described above and the expression programme was run according to the ExpiCHO™ Expression System manual. Briefly, the plasmid was amplified in *E*. *coli* DH5α and purified by the PureLink™ HiPure Plasmid Midiprep Kit (Invitrogen). CHO-S cells were subcultured for 3 generations to a density of 6–10 × 10^6^ and viability >99%. The CHO-S cells were diluted to a density of 6 × 10^6^, transfected with ExpiFectamine™ CHO Transfection Kit (Invitrogen), and cultured at 32 °C for 12 days. The supernatant was collected to purify by Ni Sepharose excel and identified by SDS-PAGE electrophoresis.

### Relative affinity determination

The relative affinities of antibodies were measured by competitive ELISA. Briefly, the concentrations of preS1 recombinant protein were diluted to 1000, 500, 100, 50, 10, 5, 1, 0.1 and 0.01 nM with PBST, the antibodies scFvD8 and scFvD8-M were adjusted to 0.5 nM and added to the solution of serial concentrations of preS1 recombinant protein (100 μL/well) to incubate at 37 °C for 1 h. Then, the reaction mixture was added to the 96-well microtiter plates, which were coated with 100 µL purified preS1 recombinant protein at concentration of 100 nM, and incubated at room temperature for 30 min. After washing, 50 µL HRP-labelled anti-SUMO antibody (1:5000 dilution in PBST) was added to the wells and then incubated at 37 °C for 30 min. Then, the enzymatic reaction was performed with TMB as the substrate and measured at 490 nm. The antibody relative affinity is the corresponding concentration of the antibody when the preS1 antigen is combined by 50%.

### Sensor construction

The whole process of sensor assembly is shown in Fig. 4. Before the electrochromic luminescence sensor was constructed, the glassy carbon electrodes (GCE, *Φ* = 3 mm) were first polished on the chamois leather with 0.3 and 0.05 μm of aluminium oxide (Al_2_O_3_) and rinsed with ultrapure water. Then, the electrodes were ultrasonicated in ultrapure water and ethanol for 5 min, and AuNPs was electrodeposited on the surface of the electrode by electrodeposition in 1% chloramic acid (HAuCl_4_) at −0.2 V for 30 s. Capture Linker (50 nM) was added dropwise to the electrode surface and incubated at 4 °C for 12 h. Finally, 6-mercapto-1-hexanol (HT) was added to the modified electrode surface and incubated at 4 °C for 2 h to block the inactive site. After each modification or reaction, every resultant electrode was rinsed with abundant PBST buffer. Each step was identified by cyclic voltammetry (CV) in 0.1 M PBS with 5 mM [Fe(CN)_6_]^3−/4−^ at the potential ranged from −0.1 V to 0.5 V with a scan rate of 50 mV/s.

### Preparation of nucleic acid labelled antibody and antigen

The coupling between the nucleic acid and the antibody or antigen was carried out using EDC/NHS reaction^40^. The general steps are as follows: 20 μL antibody or antigen (1 mg/mL) was mixed with 100 μL AbArm (1 μM) or AgArm (1 μM), respectively, and then, 50 μL of 1-ethyl-3-(3-dimethyl-aminopropyl) carbodiimide hydrochloride (EDC, 0.2 M) and 50 μL of N-hydroxysuccinimide (NHS, 0.05 M) were added together, mixed, incubated at room temperature for 2 hours, and dialyzed to obtain AbArm-labelled preS1 antibody (Ab-DNA) or AgArm-labelled preS1 antigen (Ag-DNA).

### Electrochemical detection

Human serum samples were obtained from the First Affiliated Hospital of Third Military Medical University (TMMU) with informed consents from patients or their guardians. This study was approved by the Ethics Committees of the First Affiliated Hospital of TMMU and was conducted in accordance with the Declaration of Helsinki. The electrochemical sensors were measured *via* cyclic voltammetry (CV), differential pulse voltammetry (DPV) with a CHI Instruments model 660 C electrochemical analyser in the three-electrode system (Shanghai Chen Hua Instrument, Shanghai, China). First, a series concentration of the preS1 recombinant protein standard solution in PBS buffer of 0.01 pM, 0.1 pM, 1 pM, 10 pM, 20 pM and 50 pM were prepared with four parallel controls. The DPV values of each sample were measured in the same measurement conditions. Briefly, 10 μL of the sample, 10 μL of the nucleic acid-labelled antibody, and 10 μL of the nucleic acid-labelled antigen was mixed and incubated at 37 °C for 40 minutes. Then, 5 μL was added dropwise to the surface of the modified electrode, incubated at 37 °C for 40 min and then repeatedly rinsed. DPV detection conditions: PBS solution, −0.1~0.5 V scan (5 laps/times), sweep speed 50 mV/s. Clinical samples were detected in the same manner as above.

### Data availability statement

All data in our manuscript are available.

## Electronic supplementary material

Supplementary Information

## References

[1] Franco E (2012). Hepatitis B: Epidemiology and prevention in developing countries. World journal of hepatology.

[2] Le Guillou DB, Duclos-Vallee JC, Eberle F, Capel F, Petit MA (2000). Evaluation of an enzyme-linked immunosorbent assay for detection and quantification of hepatitis B virus PreS1 envelope antigen in serum samples: comparison with two commercial assays for monitoring hepatitis B virus DNA. Journal of viral hepatitis.

[3] Nakajima E, Minami M, Ochiya T, Kagawa K, Okanoue T (1994). PreS1 deleted variants of hepatitis B virus in patients with chronic hepatitis. Journal of hepatology.

[4] Pichoud C (2000). Persistence of viral replication after anti-HBe seroconversion during antiviral therapy for chronic hepatitis B. Journal of hepatology.

[5] Liu, X. *et al*. Correlation between hepatitis B virus DNA levels and diagnostic tests for HBsAg, HBeAg, and PreS1-Ag in chronic hepatitis B. *Genetics and molecular research: GMR* **15**, 10.4238/gmr.15028282 (2016). 10.4238/gmr.15028282 27421011

[6] Lee SA (2015). Hepatitis B virus preS1 deletion is related to viral replication increase and disease progression. World journal of gastroenterology.

[7] Zhang Z (2013). A monoclonal antibody specific to the non-epitope region of hepatitis B virus preS1 contributes to more effective HBV detection. Clinical biochemistry.

[8] Jain S (2016). The Development of a Recombinant scFv Monoclonal Antibody Targeting Canine CD20 for Use in Comparative Medicine. PloS one.

[9] Wu X (2015). Fab-based bispecific antibody formats with robust biophysical properties and biological activity. mAbs.

[10] Cheng WW, Allen TM (2010). The use of single chain Fv as targeting agents for immunoliposomes: an update on immunoliposomal drugs for cancer treatment. Expert opinion on drug delivery.

[11] Miller BR (2010). Stability engineering of scFvs for the development of bispecific and multivalent antibodies. Protein engineering, design & selection: PEDS.

[12] Hur BU (2010). Development of the dual-vector system-III (DVS-III), which facilitates affinity maturation of a Fab antibody via light chain shuffling. Immunology letters.

[13] Yau KY (2005). Affinity maturation of a V(H)H by mutational hotspot randomization. Journal of immunological methods.

[14] Thakkar S, Nanaware-Kharade N, Celikel R, Peterson EC, Varughese KI (2014). Affinity improvement of a therapeutic antibody to methamphetamine and amphetamine through structure-based antibody engineering. Scientific reports.

[15] Furukawa K (2007). Strategy for affinity maturation of an antibody with high evolvability to (4-hydroxy-3-nitrophenyl) acetyl hapten. Molecular immunology.

[16] Luo J (2010). Coevolution of antibody stability and Vkappa CDR-L3 canonical structure. Journal of molecular biology.

[17] Orcutt KD (2011). Engineering an antibody with picomolar affinity to DOTA chelates of multiple radionuclides for pretargeted radioimmunotherapy and imaging. Nuclear medicine and biology.

[18] Barderas R, Desmet J, Timmerman P, Meloen R, Casal JI (2008). Affinity maturation of antibodies assisted by in silico modeling. Proceedings of the National Academy of Sciences of the United States of America.

[19] Yuan Y (2012). 3,4,9,10-perylenetetracarboxylic acid/hemin nanocomposites act as redox probes and electrocatalysts for constructing a pseudobienzyme-channeling amplified electrochemical aptasensor. Chemistry (Weinheim an der Bergstrasse, Germany).

[20] Wang J, Meng W, Zheng X, Liu S, Li G (2009). Combination of aptamer with gold nanoparticles for electrochemical signal amplification: application to sensitive detection of platelet-derived growth factor. Biosensors & bioelectronics.

[21] Cabral DG, Lima EC, Moura P, Dutra RF (2016). A label-free electrochemical immunosensor for hepatitis B based on hyaluronic acid-carbon nanotube hybrid film. Talanta.

[22] Shen Y, Shen G, Zhang Y, Zhang C, Li H (2017). A novel label-free electrochemical immunosensor based on aldehyde-terminated ionic liquid. Talanta.

[23] Chen J (2016). Amplified binding-induced homogeneous assay through catalytic cycling of analyte for ultrasensitive protein detection. Chemical communications (Cambridge, England).

[24] Gao F (2017). *Proximity hybridization trigge*red hemin/G-quadruplex formation for construction a label-free and signal-on electrochemical DNA sensor. Biosensors & bioelectronics.

[25] Hu R (2012). A proximity-dependent surface hybridization strategy for constructing an efficient signal-on electrochemical DNAzyme sensing system. Chemical communications (Cambridge, England).

[26] Xiuyuan Z, Zhihong H, Lixia W, Xiaonan L (2015). Construction of a Single Chain Variable Fragment Antibody (scFv) against Carbaryl and Its Interaction with Carbaryl. Biochemistry. Biokhimiia.

[27] Ge WS, Fan JG, Chen YW, Xu LM (2015). Expression and purification of functional HMGB1 A box by fusion with SUMO. Molecular medicine reports.

[28] Sadr V, Saffar B, Emamzadeh R (2017). Functional expression and purification of recombinant Hepcidin25 production in Escherichia coli using SUMO fusion technology. Gene.

[29] Nguyen MN, Pradhan MR, Verma C, Zhong P (2017). The interfacial character of antibody paratopes: analysis of antibody-antigen structures. Bioinformatics (Oxford, England).

[30] Heselpoth RD, Yin Y, Moult J, Nelson DC (2015). Increasing the stability of the bacteriophage endolysin PlyC using rationale-based FoldX computational modeling. Protein engineering, design & selection: PEDS.

[31] Ren K, Wu J, Zhang Y, Yan F, Ju H (2014). Proximity hybridization regulated DNA biogate for sensitive electrochemical immunoassay. Analytical chemistry.

[32] Rosenfeld R (2017). Improved antibody-based ricin neutralization by affinity maturation is correlated with slower off-rate values. Protein engineering, design & selection: PEDS.

[33] Kotlan B, Glassy MC (2009). Antibody phage display: overview of a powerful technology that has quickly translated to the clinic. Methods in molecular biology (Clifton, N.J.).

[34] Brochet X, Lefranc MP, Giudicelli V (2008). IMGT/V-QUEST: the highly customized and integrated system for IG and TR standardized V-J and V-D-J sequence analysis. Nucleic acids research.

[35] Marcatili P, Rosi A, Tramontano A (2008). PIGS: automatic prediction of antibody structures. Bioinformatics (Oxford, England).

[36] Zhang Y (2008). I-TASSER server for protein 3D structure prediction. BMC bioinformatics.

[37] Seeliger D, de Groot BL (2010). Ligand docking and binding site analysis with PyMOL and Autodock/Vina. Journal of computer-aided molecular design.

[38] Schneidman-Duhovny D, Inbar Y, Nussinov R, Wolfson HJ (2005). PatchDock and SymmDock: servers for rigid and symmetric docking. Nucleic acids research.

[39] Schymkowitz J (2005). The FoldX web server: an online force field. Nucleic acids research.

[40] Hua J (2016). Preparation and properties of EDC/NHS mediated crosslinking poly (gamma-glutamic acid)/epsilon-polylysine hydrogels. Materials science & engineering. C, Materials for biological applications.

[41] Ahangar LE, Mehrgardi MA (2017). Amplified detection of hepatitis B virus using an electrochemical DNA biosensor on a nanoporous gold platform. Bioelectrochemistry (Amsterdam, Netherlands).

[42] Heo, N. S. *et al*. Label-free electrochemical diagnosis of viral antigens with genetically engineered fusion protein. *Sensors* (*Basel, Switzerland*) **12**, 10097–10108, 10.3390/s120810097 (2012). 10.3390/s120810097 PMC3472818 23112590

[43] Liu L (2016). Multiplex electrochemiluminescence DNA sensor for determination of hepatitis B virus and hepatitis C virus based on multicolor quantum dots and Au nanoparticles. Analytica chimica acta.

