# Supplementary material for: A Highly Sensitive Detection System based on Proximity-dependent Hybridization with Computer-aided Affinity Maturation of a scFv Antibody
Source: Sci Rep. 2018 Mar 1;8:3837. doi: 10.1038/s41598-018-22111-4 (PMC5832849; doi:10.1038/s41598-018-22111-4)
Supplement: Supplementary file 1 — Supplementary Information [file 41598_2018_22111_MOESM1_ESM.docx]

**Supplementary Information**

**A Highly Sensitive Detection System based on Proximity-dependent Hybridization with Computer-aided Affinity Maturation of a scFv Antibody**

Zhiheng Wang^1^, Yan Li^2^, Wenbin Liang^1^, Junsong Zheng^2^, Shuhui Li^1^, Chuanmin Hu^1^, An Chen^1^*

^1^ Department of Clinical Biochemistry, College of Medical Laboratory, Southwest Hospital, Army Medical University (Third Military Medical University), 30 Gaotanyan Street, Shapingba District, Chongqing 400038, PR China

^2^ Department of Clinical Laboratory Science, College of Medical Laboratory, Southwest Hospital, Army Medical University (Third Military Medical University), 30 Gaotanyan Street, Shapingba District, Chongqing 400038, PR China

*Corresponding author: An Chen. E-mail：chenan@tmmu.edu.cn; Fax: +86 23 68772708


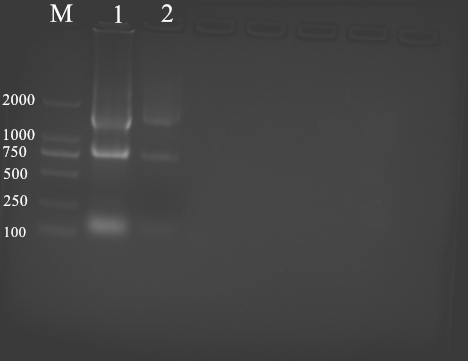


Figure S1. Total RNA extracted from mAb D8 hybridoma. Lanes: M: marker 2000; 1, 2, total RNA extracted from mAb D8 hybridoma.

(Figure S1 is the full length of the gel of Figure 1A in this manuscript)


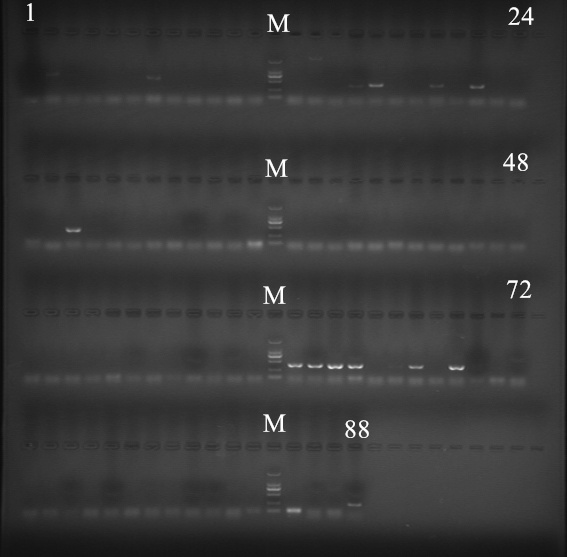


Figure S2. PCR amplification of VH and VL by 87 pairs of primers. Lanes: M: marker 2000, the descending order of the bands was 2000bp, 1000bp, 750bp, 500bp, 250bp, 100bp, respectively. The remaining lanes 1 to 45 were VH amplification results, 46 to 87 lanes were VL amplification results, the last lane 88 were the internal positive control GAPDH.

(Figure S2 is the full length of the gel of Figure 1B in this manuscript)


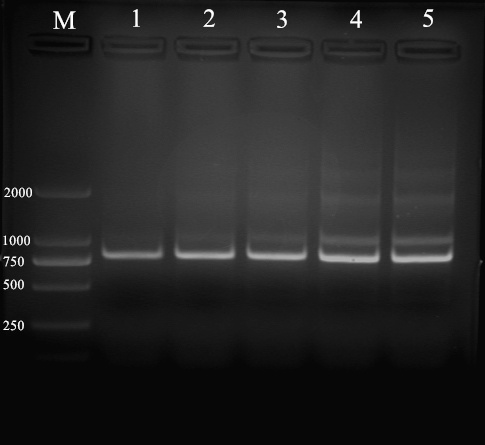


Figure S3. PCR amplification of scFv gene. Lanes: M: marker 2000; 1, 2, 3, 4, 5, the scFv fragment of anti-preS1 D8 assembled with a (G_4_S_1_)_4_ linker.

(Figure S3 is the full length of the gel of Figure 1D in this manuscript)


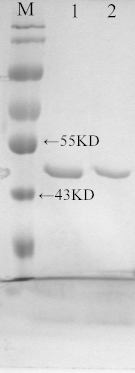


Figure S4. SDS-PAGE identification of the purified protein, which was expressed with the pET28a-SUMO vector in the BL21 host. Lanes: M, marker (beyotime P0068); 1, purified scFvD8; 2, purified scFvD8-M.

(Figure S4 is the original picture of Figure 1E in this manuscript)


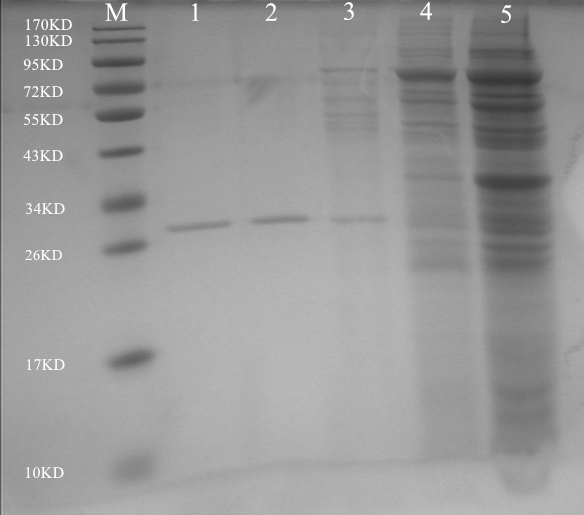


Figure S5. SDS-PAGE identification of the purified protein, which was expressed with the pcDNA3.4 vector in CHO-S cells. Lanes: M, marker (beyotime P0068); 1, purified scFvD8; 2: purified scFvD8-M; 5, CHO-S cells culture supernatant which were harvested on day 12 after plasmid transfection.

(Figure S5 is the full length of the gel of Figure 1F in this manuscript)


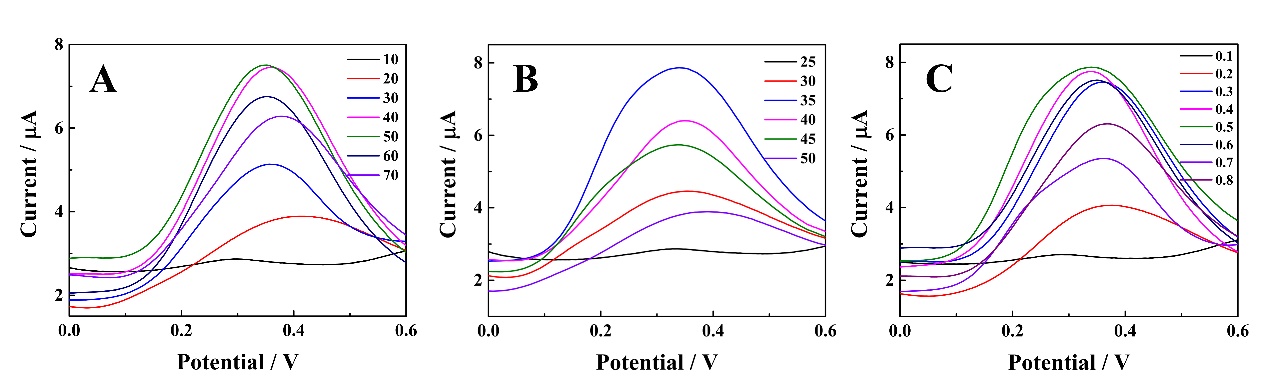


**Figure S6**. (A) Effects of the Ab-DNA/Ag-DNA concentration (10 to 70 nM) on the detection of 50 pM preS1 recombinant protein. Reaction conditions: TBE buffer (pH 7.4), 0.4 M Mg^2+^, incubation at 37 °C for 1 h. (B) Effects of temperature (25 to 50 °C) on the detection of 50 pM preS1 recombinant protein. Reaction conditions: 50 nM Ab-DNA/Ag-DNA, TBE buffer (pH 7.4), 0.4 M Mg^2+^, incubation for 1 h. (C) Effects of the Mg^2+^ concentration (0 to 0.8 M) on the detection of 50 pM preS1 recombinant protein. Reaction conditions: 50 nM Ab-DNA/Ag-DNA, TBE buffer (pH 7.4), incubation at 37 °C for 1 h.

**(Figure S6** is the main current response curves for Figure 6)


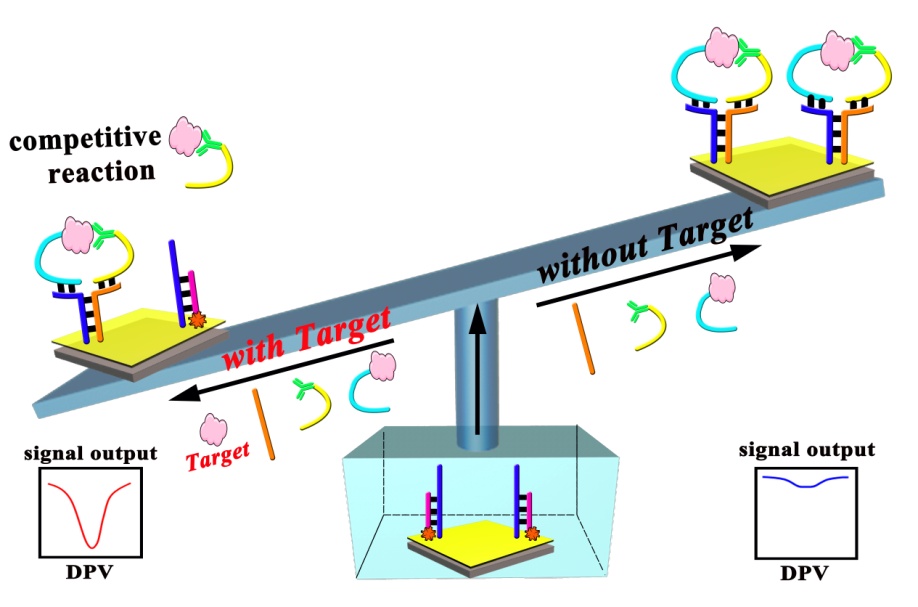


**Figure S7** The table of content (TOC) of the mechanism for the proposed biosensor system.
